# Profiling of inflammatory and anti-inflammatory cytokines in osteomyelitic bone tissue

**DOI:** 10.1515/iss-2025-0023

**Published:** 2025-09-23

**Authors:** Lara F. Lingens, Tekoshin Ammo, Tim Ruhl, Justus P. Beier

**Affiliations:** Department of Plastic Surgery, Hand Surgery – Burn Center, University Hospital RWTH Aachen, Aachen, Germany

**Keywords:** osteomyelitis, bone regeneration, cytokine profiling, microenvironment, pathogen–host interaction

## Abstract

**Objectives:**

Osteomyelitis (OM) represents a major clinical challenge in reconstructive surgery. It is characterized by chronic infection, impaired healing, and high treatment costs. Despite the high relevance, the local immune and regenerative microenvironment of infected human bone tissue remains insufficiently characterized. This study aimed to evaluate cytokine concentrations in osteomyelitic bone tissue.

**Methods:**

Bone samples were collected intraoperatively from patients with sternal, vascular, and posttraumatic OM and compared to noninfected control tissue. Cytokine concentrations of IL-6, IL-8, MCP-1, osteopontin, and SPARC were quantified in tissue homogenates by enzyme-linked immunosorbent assays (ELISA). Bacterial pathogens were identified using routine clinical microbiological diagnostics, and cytokine profiles were compared across the OM subgroups.

**Results:**

Pathogen identification revealed *Staphylococcus epidermidis* as the most common isolate in sternal OM, while vascular OM exhibited a broader spectrum including *S. epidermidis*, *Corynebacterium striatum*, *Enterobacter cloacae*, and *Proteus mirabilis*. Posttraumatic OM showed both monomicrobial and polymicrobial infections. The concentrations of the examined cytokines were significantly elevated in OM. No significant differences were observed between OM subtypes for most cytokines, except for MCP-1, which was higher in sternal compared to vascular OM. Spearman analysis revealed strong positive correlations between IL-6, IL-8, and MCP-1, indicating a coordinated inflammatory response. Osteopontin correlated significantly with inflammatory cytokines, while SPARC correlated primarily with osteopontin but not with inflammatory cytokines.

**Conclusions:**

Cytokine profiling demonstrated simultaneous activation of inflammatory and regenerative processes in OM. The preserved regenerative marker profile across OM subtypes supports individualized surgical strategies even in chronic infections. Distinct correlation patterns between inflammatory and regenerative cytokines suggest differential regulation of immune and repair mechanisms within infected bone tissue.

## Introduction

Osteomyelitis (OM) is a clinically and surgically demanding condition characterized by infection of bone tissue, often accompanied by impaired healing and long-term morbidity. Internal fixation materials, such as plates, screws, or intramedullary nails, can exacerbate infection by promoting biofilm formation, which protects pathogens from host immunity and antibiotics [1], 2]. OM may result from open fractures, surgical procedures, chronic wounds, or can be caused by hematogenous spread, and typically requires radical debridement, prolonged antibiotic therapy, and delayed reconstruction [3]. Treatment is resource-intensive and associated with high healthcare costs [4].

Despite its relevance, the molecular microenvironment within infected human bone tissue remains insufficiently characterized. Most current knowledge is derived from *in vitro* cell models or from preclinical animal studies [5]. However, these models fail to fully replicate the complexity of human OM, particularly the interplay between immune response and bone regeneration. Marriott et al. showed that osteoblasts from infected human bone express IL-6 [6], while Klosterhalfen et al. documented local and systemic cytokine release in OM patients [7]. Yet, cytokine profiling in native human OM tissue, especially in relation to the underlying pathogens, is lacking. In this study, we focused on three surgically relevant subtypes of OM: sternal, vascular, and posttraumatic OM. Each of these entities presents unique challenges. Vascular OM arises predominantly in patients with chronic wounds caused by persisting pressure (decreasing local blood flow as in pressure ulcers), diabetes, or peripheral arterial disease and is typically accompanied by compromised perfusion leading to insufficient blood and oxygen supply to the underlying bone tissue [8]. Posttraumatic OM is frequently associated with open fractures or hardware infections and often leads to segmental bone loss requiring complex reconstruction [9]. Sternal OM represents a hybrid entity, typically developing after cardiothoracic surgery. It is frequently associated with internal fixation materials (e.g., sternal wires), reduced vascular supply due to internal mammary artery harvest, thin soft tissue coverage in the sternal midline region, and sometimes even the risk of mediastinal involvement in terms of mediastinitis [10]. These three subtypes were selected to reflect a representative spectrum of etiologies, tissue perfusion status, and reconstructive needs in clinical practice.

Five cytokines involved in inflammation (interleukin-6 (IL-6), interleukin-8 (IL-8), monocyte chemoattractant protein-1 (MCP-1)) and tissue remodeling (osteopontin, SPARC (secreted protein acidic and rich in cysteine, also known as osteonectin)) were measured and analyzed across subgroups.

IL-6 and IL-8 are key proinflammatory cytokines in OM [11], 12]. MCP-1 plays a critical role in monocyte/macrophage chemotaxis and may reflect a more chronic inflammatory environment [13]. Osteopontin and SPARC, by contrast, are matricellular proteins involved in bone matrix remodeling, osteoblast function, and tissue regeneration [14], 15].

## Materials and methods

### Ethical approval

Human bone tissue samples were obtained from patients undergoing surgical procedures at the Department of Plastic Surgery, Hand Surgery, Burn Unit, University Hospital, RWTH Aachen. The tissue was collected exclusively from residual bone material that was surgically removed. The study was approved by the local ethics committee (approval number EK163/07), and all procedures were conducted in accordance with the Declaration of Helsinki.

All participants received detailed information regarding the use of their tissue for research purposes and provided written informed consent prior to inclusion. The study exclusively involved *ex vivo* analyses and did not include any interventional procedures beyond standard clinical care.

### Human tissue collection

A total of 44 bone tissue samples were analyzed, including 21 from patients with confirmed OM and 23 from uninfected controls. All samples were collected in the Department of Plastic Surgery, Hand Surgery – Burn Center at the University Hospital RWTH Aachen. The OM group consisted of six vascular, seven posttraumatic, and eight sternal OM cases. Across all OM cases, the mean patient age was 60.3 years, ranging from 23 to 88 years. This group included eight female and 13 male individuals. In the posttraumatic subgroup, the mean age was 69.7 years (range: 61–88), with three female and four male patients. The sternal subgroup had a mean age of 59.9 years (range: 41–81) and included three women and five men. The vascular subgroup had a mean age of 49.2 years (range: 23–67), with two female and four male individuals. All subgroups showed a balanced gender distribution, with a slight predominance of male patients across all groups. The control group consisted of 23 individuals with a mean age of 46.5 years (range: 1–91), including 14 women and nine men. Control samples were obtained during medically indicated surgeries in which uninfected bone tissue was removed as routine part of the standard surgical procedure. Informed consent was obtained by all 23 patients (their parents in patients <18 years of age) prior to surgery. Specifically, bone samples were collected during rib segment resections upon exposure of internal mammary artery and vein as recipient vessels for breast reconstruction (n=4), corrective osteotomies of the ulna or radius (n=2), resection arthroplasties during hand surgery (n=5), resection of accessory fingers in congenital hand deformities (n=2), nonreplantable finger parts in amputation injuries (n=4), bony shortening of toe phalanx to enable soft tissue closure following distal melanoma-related R0-resection (n=1), shaping of autologous iliac crest bone grafts, i.e., surplus bone resulting from fitting the graft (n=3), and corrective bone resections of the coracoid process to improve shoulder mobility in brachial plexus palsy and of the medial humeral epicondyle resection for release of an entrapped ulnar nerve (n=2).

Bone biopsy sites varied by group. In the posttraumatic OM group, the most frequent locations were the tibia (4 cases) and the thoracic vertebrae (2 cases). In the vascular OM group, the most common site was the os ischium, sampled in two patients. Additional samples in this group were obtained from the calcaneus, midfoot, sacrum, and toe, each in one case.

We examined pathogen distribution and local cytokine levels in bone tissue from patients with sternal, vascular, and posttraumatic OM.

Bone tissue samples were collected intraoperatively under sterile conditions. Bone pieces were separately sent to the Institute of Microbiology for standard clinical culture and antibiotic susceptibility testing as part of clinical routine work-up and transferred from the operating room to our laboratory facilities, weighed, and frozen in liquid nitrogen. Frozen bone samples were weighted and homogenized in RIPA buffer under sterile conditions at equal weights with a TissueLyser (QIAGEN Retsch) and further processed for ELISA assays. Microbiological results as documented in the patients’ medical records for pathogen classification and resistance testing were collected and correlated with experimental findings.

### Pathogen identification

Pathogen detection testing was performed using standard aerobic and anaerobic culture. Briefly, primary bone tissue was cultured on the following media: Columbia blood agar (aerobically, 37 °C), MacConkey agar (aerobically, 37 °C), Schaedler anaerobic agar (anaerobically, 37 °C), and Sabouraud dextrose agar (aerobically, 30 °C) up to 14 days. Culture plates were obtained from bioMérieux (France). Identification of bacterial isolates was performed using routine diagnostic microbiology techniques, including colony morphology and matrix-assisted laser desorption/ionization time-of-flight mass spectrometry (MALDI-TOF MS; Bruker Biotyper, Germany) and interpreted according to EUCAST clinical breakpoints. All procedures were performed at the Laboratory Diagnostic Center (LDZ) of University Hospital RWTH Aachen. The laboratory is accredited according by the German Accreditation Body (DAkkS) (D-ML-13154-03-00).

### ELISA assays

Cytokine quantification was performed using sandwich ELISA assays (human DuoSet, R&D Systems Inc., Minneapolis, USA), targeting IL-6, IL-8, MCP-1 (CCL2), osteopontin, and SPARC. Measurements were conducted according to the manufacturer’s protocols, and concentrations were normalized to tissue weight (pg/mL).

### Statistics

All statistical analyses were performed using Python (v3.11) and relevant scientific libraries. Cytokine concentrations between four study groups (sternal OM, vascular OM, posttraumatic OM, and uninfected controls) were compared using nonparametric methods due to violation of normality in at least one group, as assessed via the Shapiro–Wilk test. For pairwise comparisons between total OM and control, the Mann–Whitney U test was applied. Multiple group comparisons were performed using the Kruskal–Wallis H-test followed by Dunn’s post-hoc test. Differences were considered statistically significant at p<0.05. Spearman correlation was performed to assess the relation between inflammatory and regenerative cytokines. Diagrams were created with Inkscape and heatmaps with seaborn^©^.

## Results

Across all OM cases (n=21), *Staphylococcus epidermidis* was the most frequently detected organism. In sternal OM, it was identified both as a monoinfection and in mixed infections with *Pseudomonas aeruginosa* and *Corynebacterium tuberculostearicum*. Vascular OM cases showed a broader pathogen spectrum, including *S. epidermidis*, *Corynebacterium striatum*, *Enterobacter cloacae*, and *Proteus mirabilis*. One vascular case showed no microbiological pathogen detection, but OM was confirmed by histopathological examination. Posttraumatic OM included monoinfections with *Staphylococcus aureus*, *S. epidermidis*, *E. cloacae*, and *Klebsiella aerogenes*. Additionally, three polymicrobial infections were observed (*S. aureus* and *P. mirabilis*; *S. epidermidis* and *C. striatum*; *Corynebacterium propinquum* and *S. aureus*).

### Inflammatory response (IL-6, IL-8, MCP-1)

**Figure 1:**
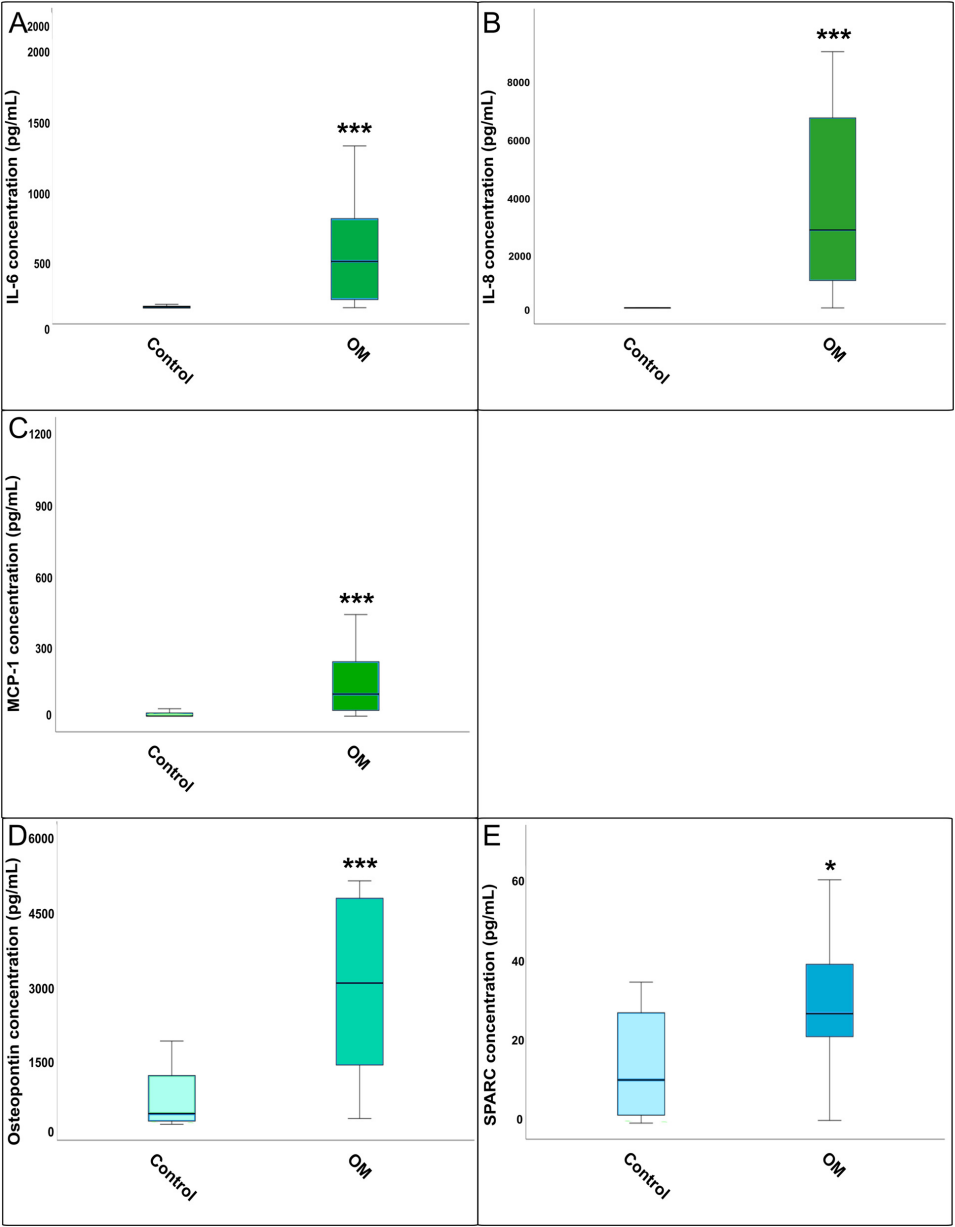
Comparison of cytokine concentration between total osteomyelitis and healthy control bone tissue: (A) IL-6, (B) IL-8, (C) MCP-1, (D) osteopontin, and (E) SPARC. Boxplots showing the concentrations of IL-6, IL-8, MCP-1, osteopontin, and SPARC measured by ELISA in osteomyelitic (OM) bone samples compared to healthy controls. Statistical differences were assessed using the Mann–Whitney U test (*=p<0.05, **=p<0.01, ***=p<0.001). Boxplots show cytokine concentrations (pg/mL) in osteomyelitic (OM) bone tissue (n=21) and uninfected controls (n=23). OM includes all subtypes combined (sternal, vascular, and posttraumatic).

**Figure 2:**
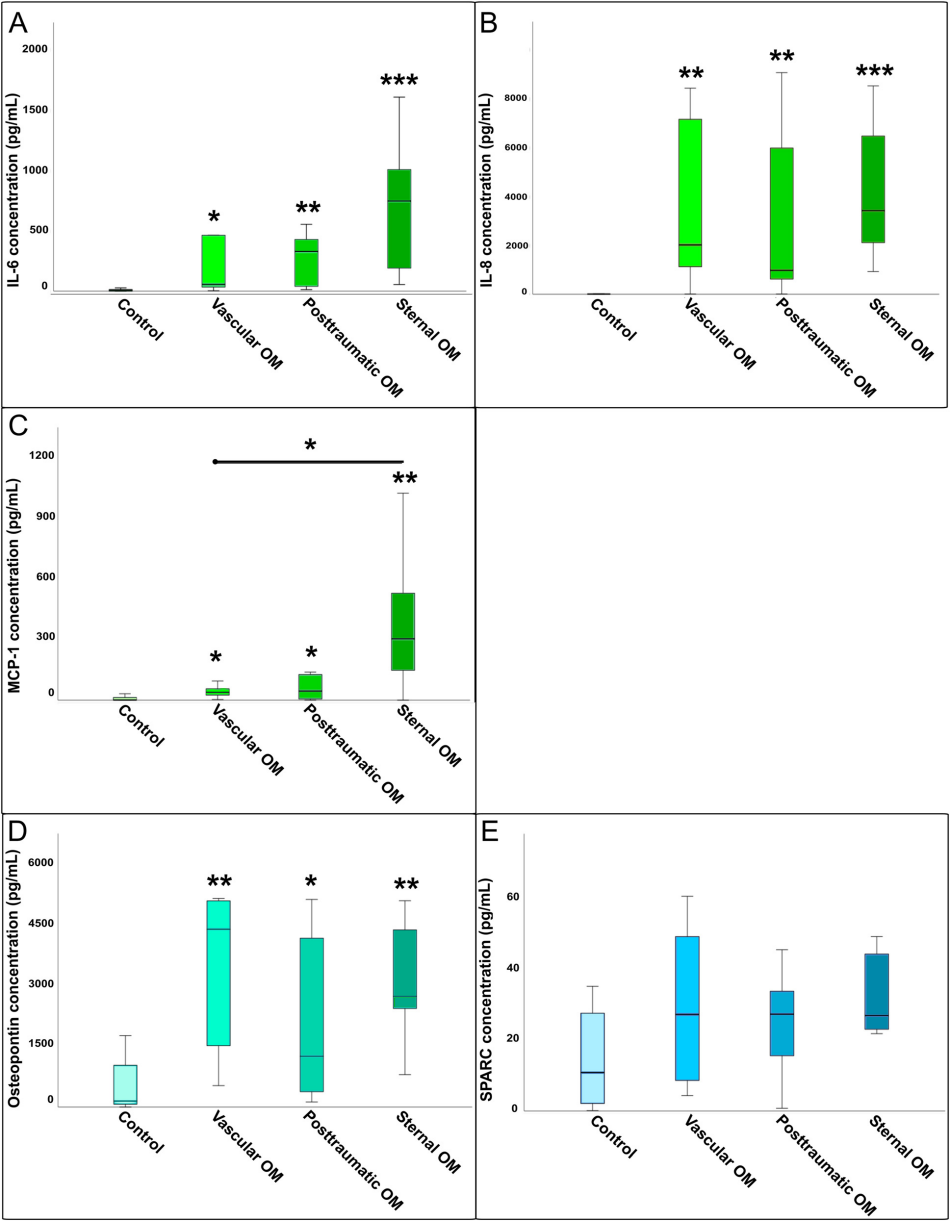
Comparison of cytokine concentrations between osteomyelitis subgroups and healthy controls: (A) Il-6, (B) IL-8, (C) MCP-1, (D) osteopontin, and (E) SPARC. Boxplots illustrating cytokine concentrations (IL-6, IL-8, MCP-1, osteopontin, and SPARC) across different osteomyelitis subtypes (sternal, vascular, posttraumatic) compared to healthy controls. Statistical differences were determined by Kruskal–Wallis tests with FDR correction (∗=p<0.05, ∗∗=p<0.01, ∗∗∗=p<0.001). Cytokine concentrations of IL-6, IL-8, MCP-1, osteopontin, and SPARC in three OM subtypes: sternal OM (n=8), vascular OM (n=6), posttraumatic OM (n=7), and uninfected controls (n=23).

IL-6 concentrations were significantly elevated in patients with OM compared to the controls (Figure 1A). Subgroup analysis revealed significantly higher IL-6 levels in all three OM subtypes vs. controls, but no significant differences occurred between the OM subgroups (Figure 2A).

IL-8 levels were also significantly increased in osteomyelitic bone tissue compared to the control (Figure 1B). The Kruskal–Wallis H-test confirmed significant differences across all groups (p<0.001). Post-hoc analysis showed that all three OM subgroups differed significantly from the control, while no significant differences were found between the OM subgroups (Figure 2B).

MCP-1 was significantly elevated in OM compared to controls (Figure 1C). The Kruskal–Wallis test indicated overall group differences for MCP-1 levels (p<0.001). Post-hoc testing showed that all OM subgroups had significantly higher MCP-1 levels compared to controls, with a significant difference also observed between sternal and vascular OM (Figure 2C).

### Regenerative capacity (osteopontin, SPARC)

Osteopontin levels were significantly higher in OM total vs. control tissue (Figure 1D), and overall group differences were confirmed by the Kruskal–Wallis H-test (p<0.001). All three subgroups showed significant increases over the control, but no significant differences were detected between the subgroups themselves (Figure 2D).

SPARC concentration was significantly increased in the OM group compared to the control (Figure 1E). However, the Kruskal–Wallis H-test found no significant differences between the four OM groups (Figure 2E).

### Pathogen-related response

**Figure 3:**
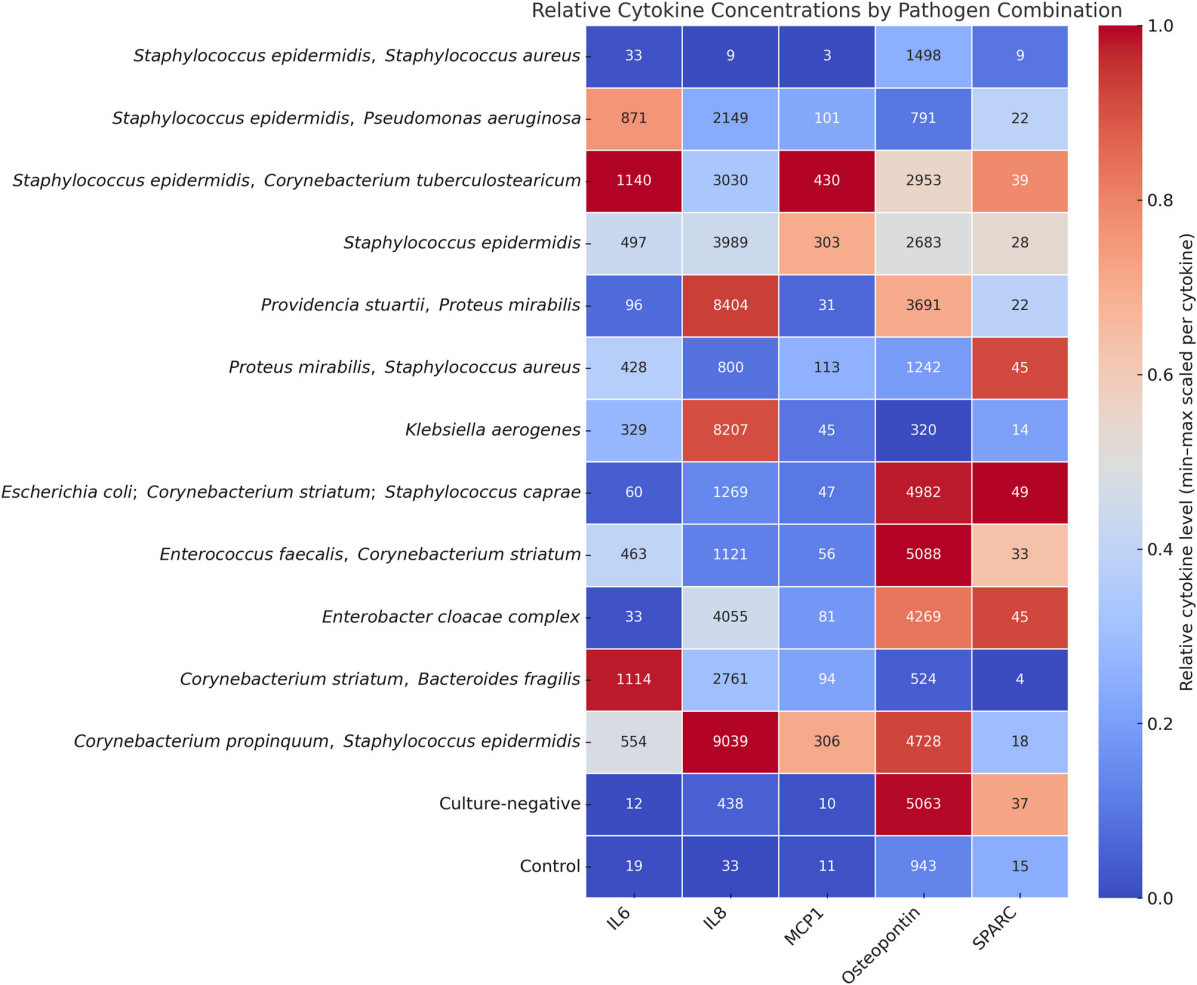
Heatmap showing the relative levels of IL-6, IL-8, MCP-1, osteopontin, and SPARC measured in osteomyelitic bone samples. Samples are grouped according to the identified pathogen species, including both monomicrobial and polymicrobial infections. Color intensity reflects the relative cytokine concentration (pg/mL). *Staphylococcus epidermidis* monoinfection (n=6) and control samples (n=23) are the only groups with more than one case; all other pathogen combinations represent individual cases (n=1).

To further explore the immunological response patterns associated with different bone infections, cytokine concentrations were compared across all confirmed pathogen combinations, including monomicrobial and polymicrobial infections. A heatmap was generated using min–max normalization per cytokine to highlight relative concentration patterns within each cytokine-pathogen combination group (Figure 3).

Notably, *S. epidermidis* and polymicrobial infections involving common skin bacteria (e.g., *S. epidermidis*, *C. striatum*) exhibited elevated IL-6 and MCP-1 levels. In contrast, pathogen combinations containing gram-negative species (e.g., *E. cloacae*, *P. mirabilis*, *Enterobacter coli*) showed high relative IL-8 and osteopontin levels.

The control group displayed uniformly low levels across all cytokines, supporting the specificity of these findings. These patterns might indicate the presence of pathogen-specific cytokine profiles, particularly distinguishing gram-positive from gram-negative infections and mono- from polymicrobial etiologies.

### Cytokine correlation

**Figure 4:**
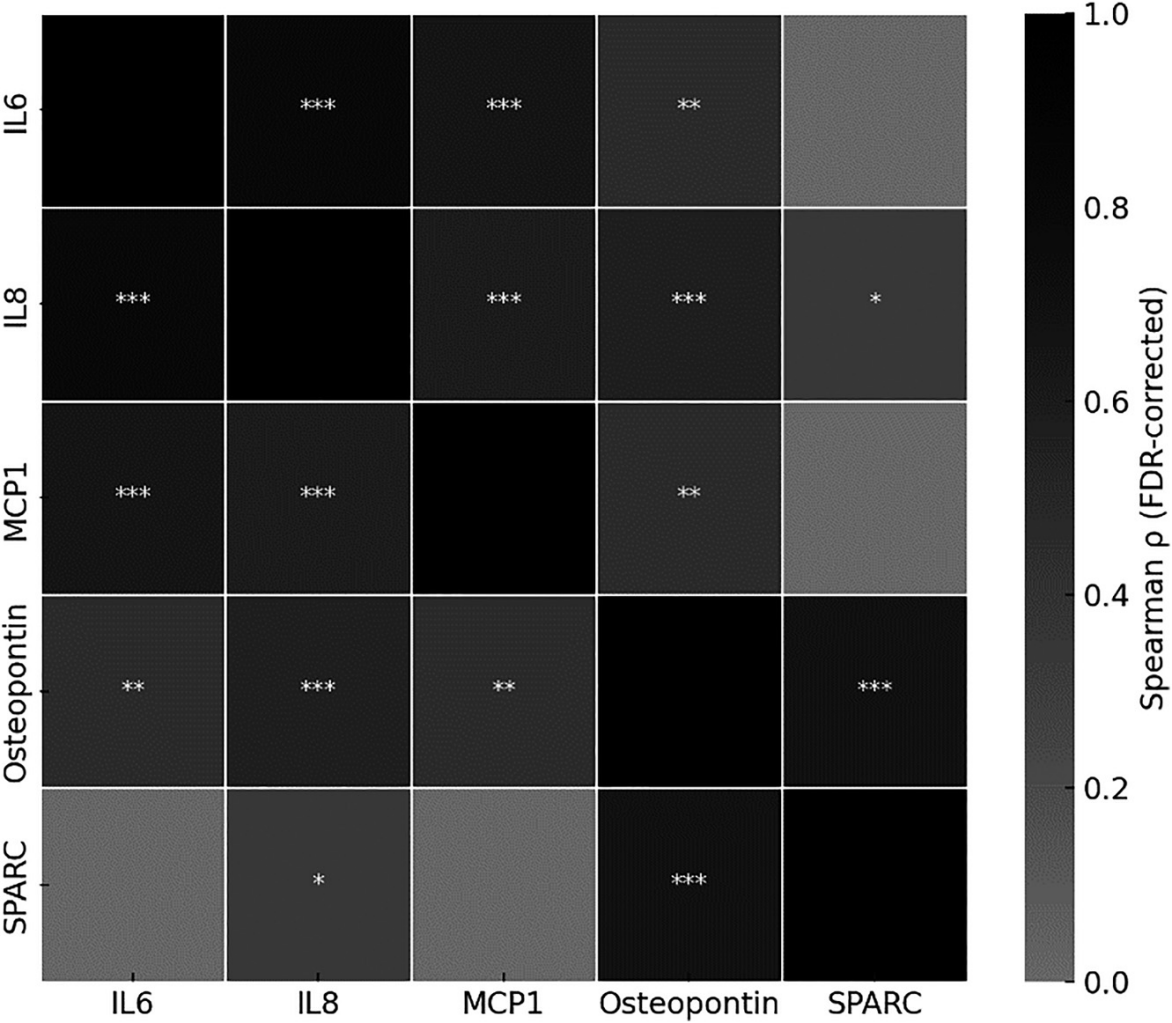
Heatmap illustrating pairwise Spearman correlation coefficients (ρ) between cytokine concentrations across all samples (n=44; OM: n=21, control: n=23). Significance levels after false discovery rate (FDR) correction are indicated as follows: ∗=p<0.05, ∗∗=p<0.01, ∗∗∗=p<0.001. Color intensity reflects the strength of the correlation.

Spearman correlation analysis revealed strong positive correlation between IL-6, IL-8, and MCP-1 (all p<0.001), suggesting a coordinated proinflammatory response. Osteopontin correlated significantly with all three inflammatory markers, while SPARC showed a strong correlation only with osteopontin (p<0.001), indicating a distinct regenerative pattern (Figure 4).

## Discussion

This study provides novel insights into the infectious and regenerative microenvironment of human osteomyelitic bone tissue and is the first cytokine-based profiling of human OM.

The observed elevations of osteopontin and SPARC in infected samples, despite ongoing inflammation, support the hypothesis that osteomyelitic bone retains regenerative capacity. This finding challenges the prevailing assumption that chronic infection necessarily precludes tissue regeneration and may influence timing and strategy in reconstructive surgery [16].

The simultaneous upregulation of inflammatory markers (IL-6, IL-8, and MCP-1) across all OM subtypes confirms a robust and coordinated innate immune response. These cytokines are well-established mediators of neutrophil and monocyte/macrophage recruitment and activation by infection [11], [12], [13]. The strong correlations between IL-6, IL-8, and MCP-1 observed in our data support this interaction and validate our methodology as sensitive to biologically relevant immune pathways.

Our findings expand upon previous work by Crisologo et al., who reported elevated serum levels of IL-6, IL-8, and MCP-1 in patients with diabetic foot osteomyelitis. While their study focused on systemic inflammatory responses [17], our analysis of cytokines directly within infected bone tissue provides complementary and more localized insight into the osteomyelitic microenvironment.

Importantly, no statistically significant differences in regenerative markers (osteopontin, SPARC) were found between the different OM subgroups (sternal, vascular, and posttraumatic), meaning that the regenerative response is preserved across OM types, regardless of anatomical site or underlying etiology. This supports the notion that all OM subtypes may possess reconstructive potential when adequately debrided and revascularized, aligning with clinical observations that successful outcomes are achievable across diverse OM presentations [18], 19].

Nonetheless, descriptive differences in inflammatory cytokine concentration suggest subtype-specific immune dynamics. For instance, higher MCP-1 levels in sternal OM may reflect a monocyte-dominated response, possibly influenced by proximity to prosthetic material [13]. This finding could also be attributed to *S. epidermidis*, which was identified in all sternal OM specimens.

Although not statistically tested, we found trends toward pathogen-specific cytokine profiles. Gram-negative and polymicrobial infections tended to present elevated IL-8 and osteopontin levels, reflecting a pronounced neutrophil-driven inflammatory reaction. Gram-positive and low-virulence pathogens such as skin commensals appeared to be associated with a higher MCP-1 concentration indicating a mononuclear-dominated immune response. These patterns are consistent with the distinct immune profiles induced by different bacterial classes: neutrophil-driven responses in gram-negative infections vs. chronic, macrophage-dominated inflammation in gram-positive or low-virulence organisms [20], [21], [22], [23], [24]. This suggests that cytokine profiling, by identifying specific patterns of inflammatory and regenerative markers in infected bone tissue, could provide insights into the severity and phase of infection. This might help determine the optimal timing for surgical intervention, guide the choice and duration of antibiotic therapy based on the dominant immune response, and predict the potential for successful tissue regeneration during reconstruction.

From a regenerative standpoint, the consistent elevation of osteopontin, and to a lesser extent SPARC, across all OM subtypes implies that matrix remodeling and osteoblast activity were not entirely suppressed in infected tissue. Osteopontin’s significant correlations with IL-6, IL-8, and MCP-1 suggested that it might have served as a mediator linking inflammation to repair. In contrast, SPARC showed a strong correlation only with osteopontin, but not with inflammatory markers, indicating a potentially more autonomous regenerative role. This differentiation could reflect separate phases or parallel pathways of bone remodeling, with SPARC possibly marking later-stage extracellular matrix stabilization [25].

Some limitations must be acknowledged. First, the sample size is relatively small, particularly within the subgroup analyses, limiting statistical power to detect differences between OM subtypes. Second, confounding factors such as patient comorbidities, duration of infection, and prior antibiotic therapy could not be fully controlled or standardized across the cohort, which may have influenced local cytokine concentrations. Third, this study is cross-sectional, capturing a single time point per patient. Longitudinal sampling would be necessary to assess cytokine dynamics over the course of infection, debridement, and healing and lastly there is a lack of matrix effect validation (e.g., cytokine spiking). Last, a limitation of this study is the difficulty in distinguishing true infection from contamination, particularly in cases involving skin commensals such as *S. epidermidis* or *Corynebacterium* spp. However, all cases in this study were histopathologically confirmed, supporting the interpretation that the detected organisms represent true pathogens. Still, the possibility of colonization or mixed flora, especially in polymicrobial infections, cannot be entirely excluded.

Despite these limitations, the present findings offer proof of concept for cytokine profiling as a clinically relevant approach to assess local immune and regenerative states in osteomyelitic bone. Future studies should integrate clinical outcomes to help validate specific markers as predictors for reconstructive success or risk of reinfection. The strong correlations between IL-6, IL-8, and MCP-1 confirm a coordinated inflammatory response, consistent with their known roles in neutrophil and monocyte recruitment [26]. The link between osteopontin and all inflammatory markers further supports its dual function in immune modulation and matrix turnover [27]. In contrast, SPARC’s independent correlation profile underscores its potential as a specific marker of regenerative matrix activity independent from the tested inflammatory markers [28].

## Conclusions

Osteomyelitic bone tissue exhibits both inflammatory activation and signs of regenerative potential. Elevated osteopontin and SPARC concentrations may serve as indicators of ongoing remodeling and tissue repair even in a poorly vascularized environment. Cytokine profiling in infected bone may support individualized surgical strategies and improve therapeutic outcomes in reconstructive procedures.
